# Coronary Artery Disease in People Living with HIV May Reflect Their Sensitivity to Inflammation Associated with Cytomegalovirus

**DOI:** 10.3390/pathogens14080822

**Published:** 2025-08-20

**Authors:** Luna-faye Veld, Shelley Waters, Silvia Lee, Anna C. Hearps, Janine Trevillyan, Ari S. Mushin, Damien Foo, Jennifer Hoy, Patricia Price

**Affiliations:** 1 Curtin Medical Research Institute, Curtin Medical School, Curtin University, Bentley, WA 6102, Australia; luna-faye.veld@outlook.com (L.-f.V.);; 2 University of Western Australia Medical School, University of Western Australia, Nedlands, WA 6008, Australia; silvia.lee@uwa.edu.au; 3 Pathwest Laboratory Medicine, Department of Microbiology and Infectious Diseases, Murdoch, WA 6150, Australia; 4 Life Sciences Discipline, Burnet Institute, Melbourne, VIC 3004, Australia; anna.hearps@burnet.edu.au; 5 Department of Infectious Diseases, Monash University, Melbourne, VIC 3004, Australia; mushinari@gmail.com (A.S.M.); jennifer.hoy@monash.edu (J.H.); 6 Department of Infectious Diseases, Peter Doherty Institute of Infection and Immunity, University of Melbourne, Melbourne, VIC 3004, Australia; janine.trevillyan@austin.org.au; 7 Department of Infectious Diseases, Austin Health, Melbourne, VIC 3004, Australia; 8 Curtin Health Nexus, Faculty of Health Sciences, Curtin University, Bentley, WA 6102, Australia; damien.foo@curtin.edu.au; 9 Department of Infectious Diseases, Alfred Hospital, Melbourne, VIC 3004, Australia

**Keywords:** cytomegalovirus, HIV, coronary artery disease, inflammatory biomarkers

## Abstract

Cytomegalovirus (CMV) is implicated in cardiovascular disease in healthy adults and after transplantation, but analyses in people living with HIV (PLWH) are difficult as almost all have CMV co-infections. Here, we address whether coronary artery disease (CAD) is associated with levels of CMV-reactive antibodies or with sensitivity to inflammation associated with CMV. PLWH stable on antiretroviral therapy (ART) with a recent diagnosis of CAD were matched with PLWH without CAD. Plasma samples stored at the time of the CAD event and 6, 12, 24 or 36 months earlier (n = 34–55 per group) were used for analyses. Antibodies reactive with a lysate from CMV infected cells were quantitated using an in-house ELISA, and inflammatory biomarkers were assessed using commercial kits. Bivariate analyses demonstrated similar levels of CMV antibodies in PLWH with and without CAD at all time points (*p* > 0.5). However, in PLWH with CAD, levels of CMV antibody correlated directly with plasma sCD14, LBP, CXCL10 and/or IL-6 at the earlier points. These correlations were not impacted by detectable plasma HIV RNA. Our findings suggest that individual differences in sensitivity to the inflammatory effects of CMV impact upon the development of CAD.

## 1. Introduction

Human cytomegalovirus (CMV) infections are commonly acquired in childhood, and the virus persists throughout life with periodic reactivations triggered by inflammatory events. Seropositivity rates are around 70% in the general population [1] and over 90% in people living with HIV (PLWH), who may have detectable CMV DNA in their plasma and exhibit end-organ disease during advanced immunodeficiency [2]. Elevated responses to CMV immediate-early-1 (IE-1) protein in PLWH suggest frequent reactivation events [3].

Meta-analyses have established associations between CMV seropositivity and cardiovascular disease (CVD) in the general population [4] and in transplant recipients [5]. The link has been attributed to direct infection of endothelial cells lining blood vessels [6] and to T-cell populations induced by CMV found in atherosclerotic plaques [7]. In our laboratory, detection of CMV DNA in saliva was a significant predictor of poor vascular health as measured by dilation of the brachial artery (flow mediated dilation; FMD) in renal transplant recipients [8]. Paradoxically, links between CMV and CVD are less clear in PLWH, despite the high incidence of CVD in PLWH [9] and their high burden of CMV. For example, in a study of 620 PLWH (586 were CMV seropositive), CMV serostatus was not associated with atherosclerosis, obstructive coronary artery disease (CAD), or plaque volumes [10]. Increased CMV antibody levels were associated with several metrics of CVD, but the effect was lost after adjustment for traditional risk factors. The identification of links between CMV and CVD may be compromised by the use of CMV-reactive antibodies as a metric of the burden of CMV, as seronegative PLWH are rare and antibody titres rise over the first year on anti-retroviral therapy (ART) [11]. Alternatively, all PLWH may have enough CMV to trigger CAD but their susceptibility may vary.

Here, we present a two-phase study of PLWH. We first sought to evaluate the association between levels of CMV-reactive antibodies and CAD immediately prior to diagnosis (T0) and 6–36 months earlier. This was achieved using stored plasma, with analyses restricted to samples collected after 12 months or more on ART. A multivariable model applied at T0 to a larger cohort from which our subset is drawn reported traditional risk factors (hypertension, age, and smoking) and previous use of the anti-HIV drug abacavir as significant predictors of CAD [12]. We investigate whether the relationship between CMV antibodies and systemic inflammation is similar in PLWH who eventually do or do not develop CAD. Our approach addresses the hypothesis that all PLWH have enough CMV to trigger CAD, but host or viral factors influence whether the pathology develops in an individual.

## 2. Methods

### 2.1. Patient Cohort

The Alfred AMI study in HIV (AASH) is a retrospective single-center case control study of CAD in PLWH treated at the Alfred Hospital, Melbourne, Australia. Cases were participants with a diagnosis of CAD defined as acute myocardial infarction, ischaemic heart disease, coronary angiogram results consistent with moderate to severe CAD, or a coronary artery bypass graft procedure. These were matched 1:2 by age (±5 years), sex and year of attendance at the Alfred Infectious Diseases Outpatient clinic with PLWH without CAD. Demographic analyses based on the entire AASH cohort addressed metrics of HIV disease, smoking history, and use of abacavir [12]. For studies of biomarkers, plasma samples were then sourced from the Victorian HIV Blood and Tissue Storage Bank (VHBTSB) from 64 AASH PLWH with CAD and 63 without CAD. These samples were drawn at the CAD event (or at selection for those without CAD) and 6, 12, 24 and/or 36 months earlier (±3 months for timepoints 6–36 months before selection). For analyses of CMV-reactive antibody herein, we excluded samples collected when an individual had been on ART ≤ 12 months (see Supplementary Table S1). HIV viraemia was defined as >200 copies HIV RNA/mL plasma. All individuals provided written informed consent for inclusion in the study and use of their samples from the VHBTSB. The project was approved by the Alfred Hospital Research and Ethics Committee (AH #205-09).

### 2.2. Plasma Inflammatory Markers

Soluble (s)CD14, lipopolysaccharide-binding protein (LBP), D-dimer, interleukin-1 receptor antagonist (IL-1Ra), vascular cell adhesion molecule-1 (VCAM-1), CXCL10, and lipoprotein-associated phospholipase A2 (LP-PLA2) were measured using customised magnetic Luminex assays (R&D systems, Minneapolis, MN, USA). Plasma interleukin-6 (IL-6) levels were measured using a high-sensitivity Quantikine ELISA (R&D systems). High-sensitivity C-reactive protein (hsCRP) was assessed batchwise using a standard clinical assay.

### 2.3. CMV-Reactive Antibody

96 half-well plates were coated with 50 μL of a lysate from human foreskin fibroblasts (HFF) infected with CMV AD169, or an uninfected HFF lysate, diluted in carbonate/bicarbonate buffer (overnight, 4 °C), washed with 0.01% TWEEN in phosphate buffered saline (PBS), and blocked with 5% bovine serum albumin (BSA)/PBS. 50 μL aliquots of sample, quality control (QC) or standards (serially diluted in 2% BSA/PBS) were added (2 h, room temperature), followed by washing. Goat Fc-specific anti-human IgG conjugated to horseradish peroxidase (Sigma–Aldrich, St Louis, MI, USA) diluted 1:4000 in 2% BSA/PBS was added and plates were incubated in the dark for one hour. After washing, 3,3′,5,5′-tetramethylbenzidine (TMB) substrate (Sigma–Aldrich) in phosphate/citrate buffer with H_2_O_2_ was added, reactions were stopped with 1 M H_2_SO_4_, and plates were read at 450 nm. Levels of antibodies reactive with CMV were interpolated from standard curves after subtraction of readings from plates coated with HFF lysate. The co-efficient of variation based on a QC sample run on every plate was 13.9%.

### 2.4. Statistical Analyses

Continuous variables were compared using non-parametric Mann–Whitney U tests and presented as median (range). Categorical variables were assessed using Fisher’s Exact tests. Continuous variables were further assessed using Spearman’s rank correlation and reported as Rho correlation coefficients. Statistical significance was defined as *p* < 0.05, but comparisons achieving 0.05 < *p* < 0.1 are noted. Multivariate logistic regressions were used to evaluate the association between multiple factors and diagnosis of CAD at each timepoint. These included age, nadir CD4, T-cell count, years on ART, receipt of abacavir at any time, and log-transformed plasma levels of CMV-reactive antibody, CD14, LBP, D-dimer, hsCRP, and IL-6. Elastic net regularisation (combining lasso and ridge to reduce overfitting) was used to identify optimum models (α = 0.5) [13]. Analyses were performed in Stata/MP version 18.5 (StataCorp LLC, College Station, TX, USA) and IBM SPSS statistics version 27 (Chicago, IL, USA).

## 3. Results

### 3.1. PLWH with and Without CAD Were Matched by Age, Sex, and Year of Attendance in the HIV Clinic

Data collected at baseline (immediately prior to selection) and 12 months earlier are summarised in Table 1. PLWH with and without CAD were matched by year of clinic attendance to minimise the impact of changes in care practices. Groups were successfully matched by age and sex at all timepoints, despite differences in group membership arising from missing samples (see Supplementary Table S1). In bivariate analyses (see Table 1), current and nadir CD4 T-cell counts, and the proportions of participants with >200 copies HIV RNA/mL were similar in PLWH with and without CAD. However, abacavir exposure was more common and the duration of ART was slightly longer in those with a CAD event.

### 3.2. A Diagnosis of CAD Did Not Align with Levels of CMV-Reactive Antibodies

At selection (T0), 91.7% with CAD and 95.8% without CAD were CMV seropositive (Table 2) with similar results at other timepoints (*p* = 0.68–1.00). At all time points, PLWH with CAD had similar levels of CMV-reactive antibodies to PLWH without CAD (*p* = 0.58–0.99).

### 3.3. CMV-Reactive Antibody Levels Correlated Directly with Selected Inflammatory Biomarkers in PLWH Who Developed CAD up to 36 Months Later

We next addressed the hypothesis that individuals who develop CAD are particularly sensitive to inflammatory consequences of persistent CMV infections. To achieve this, the cohort was stratified based on a diagnosis of CAD, and Spearman’s correlations were used to examine associations between CMV antibody levels, nadir CD4 T-cell counts, time on ART and inflammatory/cardiovascular biomarkers. Variable inverse correlations were evident between CMV antibody levels and nadir CD4 T-cell counts (0.002 < *p* < 0.55, Table 3) in PLWH with and without CAD. Levels of CMV antibodies generally increased with time on ART, but the significance of the correlation varied (0.02 < *p* < 0.75, Table 3).

**Figure 1:**
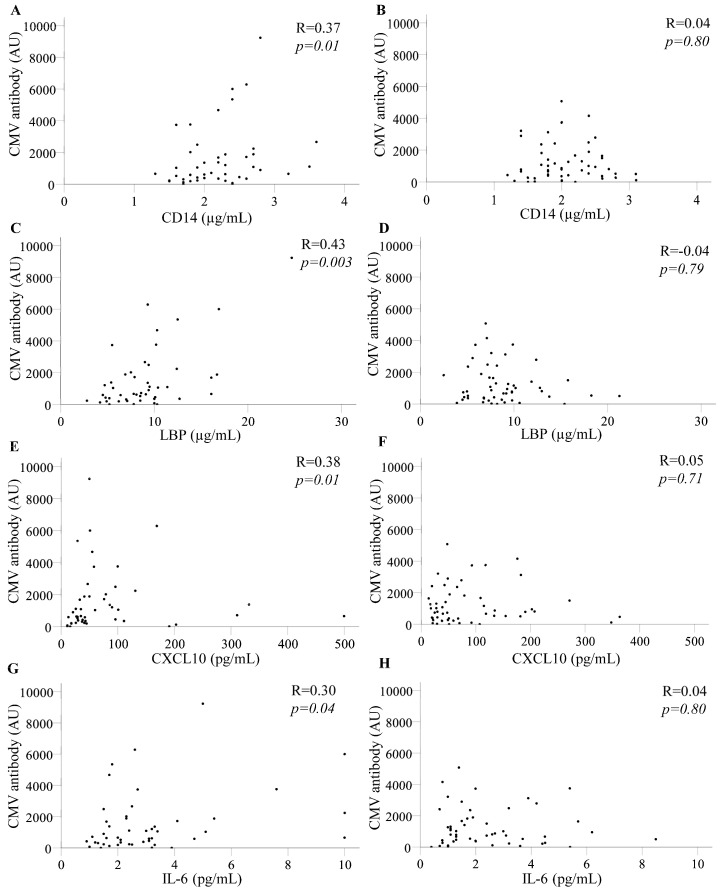
Levels of CMV-reactive antibodies correlate with inflammatory markers 12 months before a diagnosis of CAD. Scatterplots and spearman’s rank coefficients (R) are shown. Levels of CMV-reactive antibody are expressed in arbitrary units (AU). (**A**) sCD14 in PLWH who developed CAD and (**B**) sCD14 in PLWH who did not develop CAD. (**C**) LBP in PLWH who developed CAD and (**D**) LBP in PLWH who did not develop CAD. (**E**) CXCL10 in PLWH who developed CAD and (**F**) CXCL10 in PLWH who did not develop CAD. (**G**) IL-6 in PLWH who developed CAD and (**H**) IL-6 in PLWH who did not develop CAD.

When PLWH with CAD were assessed 12 months before CAD diagnosis, levels of CMV-reactive antibody correlated with plasma sCD14, LBP, CXCL10, and IL-6 (*p* = 0.003–0.04; Table 3). At 24 months, CMV antibodies correlated with CXCL10 and marginally with D-dimer, whilst at 36 months, CMV antibodies correlated with CD14, LBP, and D-dimer (Table 3). However, no biomarkers correlated with CMV antibodies in PLWH without CAD assessed 12–36 months before selection. The difference between PLWH who did and did not develop CAD 12 months later is illustrated in Figure 1. The findings suggest that CAD may be influenced by inflammation associated with CMV several years before the clinical presentation.

In an apparent contradiction to this hypothesis, significant correlations between CMV antibodies and sCD14, LBP, CXCL10, VCAM-1 and IL-6 (*p* = 0.01–0.05; Table 3) were evident in both groups 6 months before CAD event or matched timepoint. The trend was also evident at selection (T0), generally achieving 0.05 < *p* < 0.10. However, PLWH in the “no CAD” group were not followed AFTER selection, so some members may have subsequently developed CAD. This could explain the correlations observed at T0 and T-6 months. In contrast, their continued “no CAD” status over the next 12–36 months is documented at the T-12, T-24, and T-36 month assessments.

### 3.4. HIV Viraemia Did Not Explain the Correlations Between Levels of CMV-Reactive Antibody and Selected Inflammatory Biomarkers in PLWH Who Developed CAD 12–36 Months Later

When PLWH with >200 copies/mL HIV RNA were excluded (Table 4), positive correlations between CMV antibodies and sCD14, LBP, and CXCL10 remained a feature of PLWH who developed CAD 12–36 months later.

### 3.5. Associations Between CMV Antibodies and Inflammatory Markers in PLWH Who Developed CAD Did Not Reflect Higher Levels of the Markers

Here, we focus on levels of biomarkers that correlated with CMV antibodies 12–36 months before a diagnosis of CAD. In groupwise comparisons between PLWH with and without CAD, significant differences were only observed for IL-6 (T-12 and T-24 months) and sCD14 (T-24 months) (Table 5). Levels of other biomarkers were similar in PLWH with and without CAD 12, 24, and 36 months before diagnosis/selection (*p* = 0.10–0.99).

### 3.6. Multivariable Analyses Do Not Identify CMV Antibodies as Predictors of CAD

This finding was addressed further in multivariable analyses addressing CMV antibody levels plus nadir CD4 T-cell counts, time on ART, exposure to abacavir ever, and the biomarkers identified in Table 3 (sCD14, LBP, CXCL10, and IL-6). The optimal multivariable models for each time point achieved using the LASSO algorithm (Supplementary Table S2) identify abacavir as the strongest determinant of CAD, with minor effects of parameters of HIV disease and inflammatory biomarkers. CMV antibody levels were eliminated from all models by the algorithm. These results reflect findings from the broader cohort from which participants were drawn (manuscript in preparation).

## 4. Discussion

Here, we show that levels of CMV-reactive antibody measured in PLWH up to 36 months before a diagnosis of CAD are neither indicative nor predictive of CAD. Our findings align with studies reporting no association between CMV IgG titres or seropositivity and metrics of CAD in PLWH on ART. Suarez-Zdunek et al. [10] reported no significant association between CMV serostatus or CMV IgG titre and coronary artery (CA) stenosis grade or plaque volume, after adjusting for traditional CAD risk factors. Similarly, Schnittman et al. [14] found no association between CMV IgG titre and CA plaque phenotype in PLWH with moderate CVD risk. In contrast, Parinello et al. [15] reported a positive association between CMV IgG levels and CA stiffness and subclinical lesions in aviraemic women living with HIV. Knudsen et al. [16] linked CMV IgG levels with increased risk of CA calcification and increased CA thickness after adjustment for traditional CVD risk factors. Lack of consensus across published studies may arise because antibody is not a reliable metric of the CMV burden in PLWH, or because it is assessed in many different ways.

Seropositivity *per se* is used in many studies of CMV but is unhelpful in PLWH as almost all are CMV seropositive—as is evident here (Table 2) and in published studies [2,17,18]. Furthermore, antibody levels rise over the first year on ART and remain elevated compared to HIV-uninfected individuals [11]. If antibodies are used as metric of the CMV burden, extensive serial dilutions are crucial for quantitation in the high range and the antigens targeted should be considered. We quantified antibodies reactive with lysate from CMV AD169-infected human foreskin fibroblasts. This captures antibodies to all viral antigens present during active replication but may not reflect levels of antibodies to all proteins. For example, we reported associations between antibodies binding CMV IE-1 protein and arterial stiffness in renal transplant recipients, but antibodies against CMV glycoprotein-B (gB) appeared to be protective [8]. Populations of terminally differentiated effector memory T-cells reflect metrics of the burden of CMV and are expanded in PLWH stable on ART [3,19]. In future studies, it will be informative to have blood leukocytes to examine specific T-cell responses to CMV, and populations of NK cells and γδT-cells that have also been linked with an individual’s burden of CMV [20,21].

Studies quantitating CMV-reactive antibodies must also consider how much antibody corresponds to an amount of CMV sufficient to cause CAD. Some insights into susceptibility may come through associations between inflammatory markers and metrics of the burden of CMV and from assessments of circulating CMV DNA. Hastie et al. [22] described an association between CMV DNA in oral samples and plasma sCD14 and cellular markers of immune activation. Their study also showed that anti-inflammatory drugs had no significant effect on metrics of the burden of CMV, so CMV may affect inflammation rather than vice versa. This conclusion is relevant to the present study and warrants further investigation.

We found that CMV antibody levels correlated with several markers of systemic inflammation 12–36 months before CAD diagnosis, with no association in individuals who did not develop CAD. Correlations between inflammatory markers and CMV antibody levels persisted after exclusion of viraemic individuals, suggesting they are independent of active HIV replication. Our findings support the hypothesis that CMV-driven inflammation contributes to the development of CAD in PLWH, suggesting that host inflammatory response may be more critical than the CMV burden.

Viral diversity and host factors may influence susceptibility to CMV-induced systemic inflammation and viral pathogenesis. We reported a high prevalence of multi-strain infection in PLWH and renal transplant recipients, evident from sequences of viral genes from blood and saliva. Our study was based on homologues of immune-related genes encoded by CMV, with potential to alter NK function (UL18), chemokine receptors (US28) and lymphocyte activation (UL111a encoding cmvIL-10) [23,24,25]. Thus, it is possible that PLWH who developed CAD harboured strains of CMV that provoked greater inflammatory responses, creating the correlations between inflammatory biomarkers and CMV antibody levels seen 12–36 months before the diagnosis of CAD (Table 3 and Table 4).

The paradoxical associations between inflammatory markers and CMV antibody at T-6 months for those without CAD may be explained by sub-clinical cardiovascular disease or by the reality that PLWH in the “no CAD” group were not followed AFTER the selection point, so some members may have developed CAD eventually. This highlights the small sample size as a limitation of our study, as a few individuals may skew the results. The small sample size also precluded the identification of interactions in our multivariable analyses. We also acknowledge that our study was retrospective and spanned many years (1996–2018), over which time HIV treatment options changed. The removal of abacavir from current regimens may make trends clearer in future studies.

In conclusion, while CMV-reactive antibodies were not predictive of CAD, they correlated with markers of systemic inflammation selectively in PLWH who went on to develop CAD. These data support existing literature that suggests CMV-induced inflammation contributes to cardiovascular risk and disease. The study highlights limitations in using CMV antibody in PLWH. Future studies should include other metrics of the CMV burden.

## Figures and Tables

**Table 1:** PLWH with and without CAD were matched by age, sex and CD4 T-cell counts.

	CAD	No CAD	*p*-Value
*Time of diagnosis or census point*			
n	48	48	
Age (years)	52.5 (33–68)	53 (35–69)	0.90
Sex (male/female)	45/3	46/2	1.0
Nadir CD4 T-cells/µL	110 (5–618)	137 (1–494)	0.08
Current CD4 T-cells/µL	442 (8–1359)	468 (125–1131)	0.39
>200 copies/mL HIV RNA (yes/no)	9/39	7/41	0.78
Time on ART (years)	11 (1.8–28)	9.3 (1.5–22)	0.03
Ever received abacavir (yes/no)	34/14	24/24	0.06
*12 months before diagnosis or census point*			
n	47	52	
Age (years)	52 (32–68)	52 (35–68)	0.88
Sex (male/female)	45/2	50/2	1.0
Nadir CD4 T-cells/µL	110 (5–618)	132 (1–494)	0.48
Current CD4 T-cells/µL	*na*	*na*	
>200 copies/mL HIV RNA (yes/no)	12/35	10/42	0.48
Time on ART (years)	9.8 (1.1–28)	8.5 (1.3–25)	0.23
Ever received abacavir (yes/no)	29/18	20/32	0.03

Continuous data are expressed as median (range). *p* values reflect Mann–Whitney and Fisher’s Exact tests. *na* designates data not available.

**Table 2:** A diagnosis of CAD did not align with levels of antibody reactive with CMV.

	CAD	No CAD	*p*-Value
*At the time of diagnosis or census point*			
CMV serostatus (pos/neg)	44/4	46/2	0.68
CMV antibody (AU)	974 (0–8598)	838 (0–5311)	0.94
*6 months before diagnosis or census point*			
CMV serostatus (pos/neg)	48/3	51/4	1.0
CMV antibody (AU)	811 (0–8305)	603 (0–4827)	0.85
*12 months before diagnosis or census point*			
CMV serostatus (pos/neg)	45/2	50/2	1.0
CMV antibody (AU)	713 (0–9230)	800 (0–5073)	0.92
*24 months before diagnosis or census point*			
CMV serostatus (pos/neg)	40/1	50/2	1.0
CMV antibody (AU)	1043 (0–6050)	665 (0–5602)	0.58
*36 months before diagnosis or census point*			
CMV serostatus (pos/neg)	34/1	44/2	1.0
CMV antibody (AU)	894 (0–6674)	783 (0–6528)	0.99

Levels of antibodies reactive to lysate from CMV-infected cells were assessed in arbitrary units (AU) relative to a standard plasma sample and are expressed as median (range). *p* values reflect Mann–Whitney and Fisher’s Exact tests. Pos = positive, neg = negative.

**Table 3:** Levels of CMV-reactive antibodies correlate with several plasma biomarkers 12–36 months before the diagnosis of CAD.

PLWH with CAD	*Diagnosis (T0)*		*T*-*6 Months*		*T*-*12 Months*		*T*-*24 Months*		*T*-*36 Months*	
	R	*p*	R	*p*	R	*p*	R	*p*	R	*p*
n	48		51		47		41		35	
Nadir CD4 T-cells/µL	−0.09	0.55	−0.12	0.40	**−0.43**	**0.002**	*−0.28*	*0.08*	−0.24	0.16
Time on ART (years)	0.05	0.75	**0.32**	**0.02**	*0.27*	*0.07*	**0.37**	**0.02**	0.18	0.30
CD14 (μg/mL)	**0.34**	**0.02**	**0.38**	**0.01**	**0.37**	**0.01**	0.15	0.35	**0.33**	**0.05**
LBP (μg/mL)	*0.27*	*0.07*	**0.28**	**0.05**	**0.43**	**0.003**	0.22	0.17	**0.35**	**0.04**
CXCL10 (pg/mL)	*0.26*	*0.08*	**0.30**	**0.03**	**0.38**	**0.01**	**0.39**	**0.01**	0.06	0.73
IL-1RA (ng/mL)	0.06	0.69	0.11	0.46	−0.05	0.77	−0.02	0.89	0.10	0.56
D-dimer (ng/mL)	0.07	0.63	0.19	0.18	0.03	0.82	*0.27*	*0.09*	**0.39**	**0.02**
LP-PLA2 (μg/mL)	0.05	0.72	0.19	0.18	0.07	0.64	0.16	0.31	0.07	0.71
VCAM-1 (μg/mL)	0.17	0.25	**0.36**	**0.01**	0.23	0.13	0.09	0.57	0.14	0.44
hsCRP (pg/mL)	0.08	0.60	0.11	0.44	0.17	0.25	0.19	0.23	0.24	0.16
IL-6 (pg/mL)	0.22	0.13	**0.31**	**0.03**	**0.30**	**0.04**	0.25	0.11	0.18	0.31
**PLWH without CAD**	***Selection (T0)***		***T-6 months***		***T-12 months***		***T-24 months***		***T-36 months***	
n	48		55		52		52		46	
Nadir CD4 T-cells/μL	−0.14	0.33	−0.16	0.25	−0.15	0.28	−0.20	0.16	**−0.29**	**0.05**
Time on ART (years)	*0.25*	*0.08*	0.21	0.12	0.13	0.37	0.17	0.23	*0.25*	*0.10*
CD14 (μg/mL)	*0.28*	*0.06*	**0.31**	**0.02**	0.04	0.80	0.06	0.67	0.09	0.55
LBP (μg/mL)	0.23	0.11	**0.33**	**0.01**	−0.04	0.79	−0.05	0.71	−0.08	0.59
CXCL10 (pg/mL)	*0.27*	*0.06*	**0.31**	**0.02**	0.05	0.71	0.09	0.54	0.13	0.39
IL-1RA (ng/mL)	−0.07	0.66	0.00	0.98	0.08	0.56	0.04	0.76	−0.08	0.60
D-dimer (ng/mL)	0.08	0.58	0.02	0.90	−0.11	0.44	−0.09	0.55	−0.17	0.26
LP-PLA2 (µg/mL)	−0.08	0.57	−0.08	0.55	−0.03	0.84	−0.02	0.88	0.03	0.84
VCAM-1 (μg/mL)	0.22	0.13	**0.29**	**0.04**	0.09	0.55	0.13	0.37	0.04	0.79
hsCRP (pg/mL)	0.11	0.45	0.10	0.45	−0.03	0.83	−0.04	0.77	−0.07	0.63
IL-6 (pg/mL)	0.14	0.34	0.05	0.70	0.04	0.80	0.18	0.19	−0.05	0.75

PLWH with and without a diagnosis of CAD were analysed separately. Plasma levels of inflammatory and cardiac biomarkers were aligned with levels of antibodies reactive with CMV-infected cells (in AU) using Spearman’s rank correlations. Rho and *p*-values are shown. Correlations achieving *p* < 0.05 are in bold font, and 0.05 < *p* < 0.10 is marked in italics. Assessments were made at diagnosis/selection and using samples archived 6, 12, 24, and 36 months earlier.

**Table 4:** Levels of CMV-reactive antibodies in plasma correlated with CD14, LBP, and CXCL10 12–36 months before the diagnosis of CAD, when PLWH with >200 copies/mL HIV RNA were excluded.

PLWH with CAD	*T*-*12 Months*		*T*-*24 Months*		*T*-*36 Months*	
	R	*p*	R	*p*	R	*p*
n	35		33		27	
Nadir CD4 T-cells/μL	−0.24	0.16	−0.24	0.18	−0.11	0.59
Time on ART (years)	**0.34**	**0.05**	**0.38**	**0.03**	0.26	0.19
sCD14 (μg/mL)	**0.39**	**0.02**	0.02	0.90	**0.39**	**0.05**
LBP (μg/mL)	**0.60**	**<0.001**	0.25	0.17	**0.51**	**0.007**
CXCL10 (pg/mL)	**0.35**	**0.04**	**0.45**	**0.01**	0.27	0.18
IL-6 (pg/mL)	0.18	0.30	0.23	0.19	*0.34*	*0.08*
PLWH without CAD	*T*-*12 months*		*T*-*24 months*		*T*-*36 months*	
n	42		42		38	
Nadir CD4 T-cells/μL	−0.13	0.42	−0.22	0.17	−0.17	0.31
Time on ART (years)	0.06	0.71	0.06	0.71	0.16	*0.33*
sCD14 (μg/mL)	0.03	0.87	0.21	0.19	0.03	0.86
LBP (μg/mL)	−0.13	0.42	−0.03	0.87	−0.19	0.26
CXCL10 (pg/mL)	0.09	0.55	*0.26*	*0.10*	0.14	0.39
IL-6 (pg/mL)	−0.03	0.87	0.24	0.13	−0.07	0.66

PLWH with and without a diagnosis of CAD were analysed separately. Plasma levels of inflammatory and cardiac biomarkers identified in Table 1 were aligned with levels of CMV-reactive antibodies (in AU) using Spearman’s rank correlations. Rho and *p*-values are shown. Correlations achieving *p* < 0.05 are in bold font, and 0.05 < *p* < 0.10 is marked in italics.

**Table 5:** Levels of inflammatory biomarkers that correlate with CMV-reactive antibodies in PLWH who developed CAD do not align uniformly with CAD diagnosis in groupwise comparisons.

	CAD	No CAD	*p*-Value
*12 months before diagnosis or selection*			
n	47	52	
sCD14 (μg/mL)	2.2 (1.3–3.6)	2 (1.2–3.1)	0.27
LBP (μg/mL)	8.6 (2.8–25)	8 (2.5–21)	0.58
CXCL10 (pg/mL)	45 (11–500)	51 (14–364)	0.27
IL-6 (pg/mL)	2.5 (0.9–10)	1.8 (0.4–8.5)	**0.02**
*24 months before diagnosis or selection*			
n	41	52	
sCD14 (μg/mL)	2.3 (1.5–4.0)	1.9 (1.2–3.9)	**0.004**
LBP (μg/mL)	8.7 (2.8–32)	7.5 (2.3–16)	*0.10*
CXCL10 (pg/mL)	49 (18–500)	52 (15–330)	0.99
IL-6 (pg/mL)	2.2 (0.9–10)	1.7 (0.6–10)	**0.02**
*36 months before diagnosis or selection*			
n	35	46	
sCD14 (μg/mL)	2.1 (1.4–3.1)	2.1 (1.0–3.6)	0.30
LBP (μg/mL)	8.2 (3.3–18)	7.5 (2.3–33)	0.29
CXCL10 (pg/mL)	49 (12–391)	52 (14–200)	0.69
IL-6 (pg/mL)	1.6 (0.5–10)	1.7 (0.6–10)	0.37

Data are presented as median (range) and analyses used Mann–Whitney tests.

## Data Availability

De-identified data are available on request.

## Supplementary Materials

The following supporting information can be downloaded at: https://www.mdpi.com/article/10.3390/pathogens14080822/s1, Table S1: Participant sample selection; Table S2: Multivariable analyses do not identify CMV antibodies as predictors of CAD.
