# Supplementary material for: Coronary Artery Disease in People Living with HIV May Reflect Their Sensitivity to Inflammation Associated with Cytomegalovirus
Source: Pathogens. 2025 Aug 20;14(8):822. doi: 10.3390/pathogens14080822 (PMC12389153; doi:10.3390/pathogens14080822)
Supplement: Supplementary file 1 [file pathogens-14-00822-s001.zip › pathogens-3758781-supplementary.pdf]

Supplementary Table S1: Participant sample selection

	PLWH with CAD					PLWH without CAD				
n	64					63				
	Samples available from +/-3 months of given timepoint									
T	0	-6	-12	-24	-36	0	-6	-12	-24	-36
n	52	54	48	42	36	51	60	57	58	51
	Remove duplicate samples									
n	51	53	48	42	36	51	58	57	58	50
	Remove samples with no ART data available									
n	50	53	48	42	36	51	58	57	58	50
	Remove samples with ART <12 months									
n	48	51	48	41	35	48	55	52	52	46
	Remove samples with HIV RNA > 200 copies / mL or missing viral load data									
n	39	38	35	33	27	41	47	42	42	38

Selection of stored plasma samples for study inclusion. PLWH=people living with HIV. CAD =coronary artery disease. ART=anti-retroviral therapy. T = timepoint in months.

Supplementary Table S2: Multivariable analyses do not identify CMV antibodies as predictors of CAD

	Odds ratio	P	95% CI	
			lower	upper
**T -12 months;** pseudo R^2=0.07, N=99, p=0.018				
IL-6	2.00	**0.042**	1.03	3.90
abacavir ever	2.27	0.053	0.99	5.21
**T -24 months;** pseudo R^2=0.17, N=92, p=0.015				
sCD14	13.9	0.056	0.94	207
LBP	1.45	0.561	0.41	5.13
CXCL10	1.00	0.211	0.99	1.00
IL-6	1.39	0.431	0.61	3.19
Years on ART	1.01	0.774	0.93	1.10
abacavir ever	4.81	**0.007**	1.52	15.2
**T -36 months;** pseudo R^2=0.12, N=81, p=0.027				
sCD14	2.00	0.571	0.18	22.1
LBP	1.76	0.463	0.39	8.00
Years on ART	1.04	0.367	0.95	1.14
Nadir CD4 T-cells	1.00	0.237	0.99	1.00
abacavir ever	3.54	**0.019**	1.24	10.1

Multivariate logistical regression analyses identifying factors associated with a diagnosis of CAD were performed using data obtained at 12-36 months before diagnosis/recruitment. Analyses began with all factors included (age, nadir CD4 T-cell count, years on ART and receipt of abacavir at any time, plus log transformed data describing plasma levels of CMV-reactive antibody, CD14, LBP, CXCL10 and IL-6. Elastic net-guided Model (LASSO and Ridge Regressions; α = 0.5) were used to create optimal models.
